# From Data Completion to Problems on Hypercubes: A Parameterized Analysis of the Independent Set Problem

**DOI:** 10.1007/s00453-025-01354-4

**Published:** 2025-11-17

**Authors:** Eduard Eiben, Robert Ganian, Iyad Kanj, Sebastian Ordyniak, Stefan Szeider

**Affiliations:** 1 Department of Computer Science, Royal Holloway, University of London, Egham, UK; 2 Algorithms and Complexity Group, TU Wien, Vienna, Austria; 3 School of Computing, DePaul University, Chicago, USA; 4 School of Computing, University of Leeds, Leeds, UK

**Keywords:** Parameterized complexity, Data completion, Diversity, Clustering, Independent set problem on hypercubes, First Order Logic model checking

## Abstract

Several works have recently investigated the parameterized complexity of data completion problems, motivated by their applications in machine learning, and clustering in particular. Interestingly, these problems can be equivalently formulated as classical graph problems on induced subgraphs of powers of partially-defined hypercubes. In this paper, we follow up on this recent direction by investigating the Independent Set problem on this graph class, which has been studied in the data science setting under the name Diversity. We obtain a comprehensive picture of the problem’s parameterized complexity and establish its fixed-parameter tractability w.r.t. the solution size plus the power of the hypercube. Given that several such First Order Logic (FO) definable problems have been shown to be fixed-parameter tractable on the considered graph class, one may ask whether fixed-parameter tractability could be extended to capture all FO-definable problems. We answer this question in the negative by showing that FO model checking on induced subgraphs of hypercubes is as difficult as FO model checking on general graphs.

## Introduction

Recently, there has been an increasing interest in studying the parameterized complexity of clustering problems motivated by their applications in machine learning [2–6, 17, 19, 21, 26, 27, 31, 40, 41], particularly their applications to fundamental clustering problems [1, 29, 42, 45]. In many of these clustering problems, we are given a set of *d*-dimensional vectors over the Boolean/binary domain, where the vectors are regarded as rows of a matrix. It is worth noting that due to the applications of such problems in incomplete-data settings, a number of past works on the topic also studied settings where some of the entries in these vectors are unknown [8–10, 17, 19, 21, 22, 30, 31, 38]. The objective is to determine if these vectors (or, in the incomplete-data setting, their completions) satisfy some desirable clustering properties. Examples of such properties include admitting a partitioning into *k* clusters each of diameter (or radius) at most *r* (for some given $k, r \in \mathbb {N}$), or admitting a *k*-cluster of diameter (or radius) at most *r*, where the distance under consideration is typically the Hamming distance [12, 16, 19, 21, 24, 33–36]; here, a *k*-cluster of diameter *r* is a set of *k* points which have pairwise distance of at most *r*.

As it turns out, many of these well-studied clustering problems can be formulated as classical graph problems on induced subgraphs of powers of the hypercube graph. For instance, finding a cluster of diameter at most $r \in \mathbb {N}$, for a given *r*, is equivalent to the Clique problem defined on the subgraph of the *r*-th power of the hypercube that is induced by the subset of hypercube vertices corresponding to the given input vectors. Similarly, partitioning the set of vectors into *k* clusters each of diameter at most *r*, for some given $r, k \in \mathbb {N}$, is equivalent to the partitioning into *k* cliques problem on the same graph class, whereas partitioning the set of vectors into clusters, each of radius at most *r* with respect to some vector in the set, is equivalent to the *k*-dominating set problem on the same graph class described above. We remark that, to the best of our knowledge, this graph class is not a subclass of commonly studied graph classes and has not been considered in previous works pertaining to algorithmic upper or lower bounds for graph-theoretic problems.

*Contribution.* In this paper, we study the parameterized complexity of another classical graph problem defined on induced subgraphs of powers of the hypercube: the Independent Set problem. In the context of data analytics, the problem arises when studying the “diversity” of a given set of vectors, a notion that can be viewed as the opposite of minimising, i.e., maximising, the number of clusters in a cluster partitioning of the set of vectors (in fact, in the area of data analytics this problem is studied directly under the nomenclature *diversity* or dispersion [11, 32, 46]). More precisely, motivated by the aforementioned extensive interest in the analysis of incomplete data, we focus on the more general incomplete data setting. We refer to this problem as Pow-Hyp-IS-Completion: given a set of Boolean vectors with some missing entries and integers *k* and *r*, the goal is to complete the missing entries so that the resulting set of vectors contains a subset *S* of *k* vectors such that the Hamming distance between each pair is at least $r+1$ (or to correctly determine that such a set does not exist).

The main contribution of this paper is a complete characterisation of the parameterized complexity of Pow-Hyp-IS-Completion w.r.t. the two parameters *k* and *r*: we provide a fixed-parameter algorithm for Pow-Hyp-IS-Completion when parameterized by $k+r$, and complement this positive result with intractability results for the cases where any of these two parameters is dropped. In particular, we show that the problem is NP-complete already for $r=2$—that is, the problem is paraNP * -hard* parameterized by *r*, and W[1]-hard parameterized by *k* alone. Interestingly, the FPT result shows that the parameterized complexity of the problem is independent of any restrictions on the number or the structure of the missing entries in the input vectors—contrasting many of the previous results on clustering incomplete data [19, 21, 30, 31]. We remark that even the fixed-parameter tractability of the problem in the complete data setting (i.e., where all entries are known) is non-obvious, but follows as an immediate corollary of our result.

For our final contribution, we revisit the observation that several of the complete-data clustering problems recently considered in the literature (e.g., see [19, 21]) reduce to well-known graph problems on the class of induced subgraphs of powers of the hypercube. Since it was shown that all of these graph problems are fixed-parameter tractable when restricted to this graph class and the graph problems are expressible in First Order Logic (FO), a natural question to ask is whether these FPT results can be generalised to any graph problem expressible in FO logic. We resolve this question in the negative.

*Related Work.* The problem of computing the diversity of a data set, which forms the underpinning of our study of Pow-Hyp-IS-Completion, has been studied in a variety of different contexts and settings. For instance, Ceccarello, Pietracaprina, Pucci and Upfal studied approximation algorithms for the problem [11]. Gawrychowski, Krasnopolsky, Mozes, and Weimann obtained a linear-time algorithm for the problem when the data set is represented as a tree [32], improving upon the previous polynomial-time algorithm of Bhattacharya and Houle [7]. Sacharidis, Mehta, Skoutas, Patroumpas and Voisard provided heuristics for dynamic versions of the problem [46].

More broadly, there is extensive work on problems arising in the context of incomplete data. Hermelin and Rozenberg [39] studied the Closest String with Wildcards problem, which can be seen as the problem of finding a data completion and a center to a minimum-radius cluster containing all the data points. Koana, Froese and Niedermeier [40] recently revisited the earlier work of Hermelin and Rozenberg [39] and obtained, among other results, a fixed-parameter algorithm for that problem parameterized by the radius plus the maximum number of missing entries per row; see also the related work of the same authors [41]. Eiben et al. considered a number of different clustering problems in the presence of incomplete data [18, 19], and a subset of these authors previously investigated the fundamental Matrix Completion problem in the same setting [30]. The parameterized complexity of *k*-means clustering on incomplete data was investigated by Eiben et al. [17] and Ganian et al. [31].

*Parts of this paper appeared in a preliminary and shortened form in the Proceedings of IPEC 2023, 18th International Symposium on Parameterized and Exact Computation*[20].

## Preliminaries

Problem Terminology and Definition

Let $\vec {a}$ and $\vec {b}$ be two vectors in $\{0,1,{\small \square }\}^d$, where ${\small \square }$ is used to represent coordinates whose value is unknown (*i.e.*, missing entries). We denote by $\Delta (\vec {a},\vec {b})$ the set of coordinates in which $\vec {a}$ and $\vec {b}$ are guaranteed to differ, *i.e.*, $\Delta (\vec {a},\vec {b})=\{\,i \;{|}\;(\vec {a}[i]=1 \wedge \vec {b}[i]=0)\vee (\vec {a}[i]=0 \wedge \vec {b}[i]=1) \,\}$, and we denote by $\delta (\vec {a},\vec {b})$ the *Hamming distance* between $\vec {a}$ and $\vec {b}$ measured only between known entries, *i.e.*, $|\Delta (\vec {a},\vec {b})|$. Moreover, for a subset $D' \subseteq [d]$ of coordinates, we denote by $\vec {a}[D']$ the vector $\vec {a}$ restricted to the coordinates in $D'$.

Let $M\subseteq \{0,1\}^d$ and let $[d]=\{1,\dots ,d\}$. For a vector $\vec {a} \in M$ and $t \in \mathbb {N}$, we denote by $N_{t}({\vec {a}})$ the *t-Hamming neighbourhood* of $\vec {a}$, *i.e.*, the set $\{\,\vec {b} \in M \;{|}\;\delta (\vec {a},\vec {b})\le t\,\}$ and by $N_{t}({M})$ the set $\bigcup _{\vec {a} \in M}N_{t}({\vec {a}})$. We say that $M^*\subseteq \{0,1\}^d$ is a *completion* of $M\subseteq \{0,1,{\small \square }\}^d$ if there is a bijection $\alpha : M \rightarrow M^*$ such that for all $\vec {a}\in M$ and all $i\in [d]$ it holds that either $\vec {a}[i]={\small \square }$ or $\alpha (\vec {a})[i]=\vec {a}[i]$.

We now proceed to give the formal definition of the problem under consideration:

**Table:** 

Pow-Hyp-IS-completion	
Input	A set *M* with elements from $\{0,1,{\small \square }\}^d$ and $k, r \in \mathbb {N}$.
Question	Is there a completion $M^{*}$ of *M* and a subset *S* of $M^{*}$ with $|S|= k$ such that, for any two vectors $a, b \in S$, we have $\delta (a, b) \ge r+1$?

Observe that in a matrix representation of the above problem, we can represent the input matrix as a *set* of vectors where each row of the matrix corresponds to one element in our set.

We remark that even though the statements are given in the form of decision problems, all tractability results presented in this paper are constructive and the associated algorithms can also output a solution (when it exists) as a witness, along with the decision. In the case where we restrict the input to vectors over $\{0,1\}^d$ (*i.e.*, where all entries are known), we omit “-Completion” from the problem name.

*Parameterized Complexity*

The basic motivation behind parameterized complexity is to find a parameter that describes the structure of the problem instance such that the combinatorial explosion can be confined to this parameter. More formally, a *parameterized problem* *Q* is a subset of $\Omega ^{*} \times \mathbb {N}$, where $\Omega$ is a fixed finite alphabet. Each instance of *Q* is a pair $(I, \kappa )$, where $\kappa \in \mathbb {N}$ is called the *parameter*. A parameterized problem *Q* is *fixed-parameter tractable* (FPT) [13, 14, 25], if there is an algorithm, called an FPT*-algorithm*, that decides whether an input $(I, \kappa )$ is a member of *Q* in time $f(\kappa ) \cdot |I|^{\mathcal {O}(1)}$, where *f* is a computable function and |*I*| is the input instance size. The class FPT denotes the class of all fixed-parameter tractable parameterized problems.

A parameterized problem *Q* is FPT*-reducible* to a parameterized problem $Q'$ if there is an algorithm, called an FPT*-reduction*, that transforms each instance $(I, \kappa )$ of *Q* into an instance $(I', \kappa ')$ of $Q'$ in time $f(\kappa )\cdot |I|^{\mathcal {O}(1)}$, such that $\kappa ' \le g(\kappa )$ and $(I, \kappa ) \in Q$ if and only if $(I', \kappa ') \in Q'$, where *f* and *g* are computable functions. Based on the notion of FPT-reducibility, a hierarchy of parameterized complexity, *the* W*-hierarchy* $=\bigcup _{t \ge 0} {{{\hbox {\text {\textsf {W}}}}}}{{ {[t]}}}$, where ${{{\hbox {\text {\textsf {W}}}}}}{{ {[t]}}} \subseteq {{{\hbox {\text {\textsf {W}}}}}}{{ {[t+1]}}}$ for all $t \ge 0$, has been introduced, in which the 0-th level W[0] is the class FPT . The notions of hardness and completeness have been defined for each level W[*i*] of the W-hierarchy for $i \ge 1$ [13, 14]. It is commonly believed that ${{{\hbox {\text {\textsf {W}}}}}}{{ {[1]}}} \ne {\hbox {\text {\textsf {FPT}}}}$ (see [13, 14]). The W[1]-hardness has served as the main working hypothesis of fixed-parameter intractability. A problem is paraNP * -hard* if it is NP-hard for a constant value of the parameter [25].

*Sunflowers*

A *sunflower* in a set family $\mathcal {F}$ is a subset $\mathcal {F}' \subseteq \mathcal {F}$ such that all pairs of elements in $\mathcal {F}'$ have the same intersection.

### Lemma 1

([23, 25]) Let $\mathcal {F}$ be a family of subsets of a universe *U*, each of cardinality exactly *b*, and let $a \in \mathbb {N}$. If $|\mathcal {F}|\ge b!(a-1)^{b}$, then $\mathcal {F}$ contains a sunflower $\mathcal {F}'$ of cardinality at least *a*. Moreover, $\mathcal {F}'$ can be computed in time polynomial in $|\mathcal {F}|$.

## The Parameterized Complexity of Pow-Hyp-IS-Completion

Our aim for Pow-Hyp-IS-Completion is to establish fixed-parameter tractability parameterized by $k+r$ (*i.e.*, regardless of the structure or number of missing entries). As our first step, we show that all rows in an arbitrary instance (*M*, *k*, *r*) can be, w.l.o.g., assumed to contain at most $\mathcal {O}(k\cdot r)$ many ${\small \square }$’s.

Next, we observe that if *M* is sufficiently large and the *r*-Hamming neighbourhood of each vector is upper-bounded by a function of $k+r$, then—since the number of ${\small \square }$’s is bounded—(*M*, *k*, *r*) is a YES-instance. The argument here is analogous to the classical argument showing that Independent Set is trivial on large bounded-degree graphs.

On a high level, we would now like to find and remove an “irrelevant vector” from *M*—since here the number of ${\small \square }$’s on *every* row is bounded, any instance reduced in this way to only contain a bounded number of vectors can be solved via a brute-force fixed-parameter procedure. However, finding an irrelevant vector is rather challenging, primarily because the occurrence of ${\small \square }$’s is not restricted. Instead, we develop a more powerful set representation $\mathcal {F}'$ for vectors in the instance which also uses elements to keep track of the presence of ${\small \square }$’s in the neighbours of $\vec {v}$. We can then apply the Sunflower Lemma to find a sufficiently-large sunflower in $\mathcal {F}'$, and in the core of the proof we argue that (1) such a sunflower consists of at most a bounded number of “important petals” (which can be identified in polynomial time), and (2) any petal that is not important represents an irrelevant vector.

### Dealing with Unstructured Missing Data

In this subsection, we design an algorithm for Pow-Hyp-IS-Completion which remains efficient even when the number and placement of unknown entries is not explicitly restricted on the input.

We begin with a simple lemma that allows us to deal with vectors (*i.e.*, rows) with a large number of missing entries. For brevity, let a *k*-diversity set be a set containing *k* vectors which have pairwise Hamming distance at least $r+1$.

#### Lemma 2

Let $\mathcal {I}=(M,k,r)$ be an instance of Pow-Hyp-IS-Completion where $k\ge 1$ and let $\vec {v}\in M$ be a vector containing more than $(k-1)\cdot (r+1)$-many ${\small \square }$’s. Then $\mathcal {I}$ is a YES-instance if and only if $\mathcal {I}'=(M\setminus \{\vec {v}\},k-1,r)$ is a YES-instance. Moreover, a completion and *k*-diversity set for $\mathcal {I}$ can be computed from a completion and $(k-1)$-diversity set for $\mathcal {I}'$ in linear time.

#### Proof

The forward direction is trivial: for any completion $M^{*}$ of *M* and *k*-diversity set *S* in $M^{*}$, we can obtain a $(k-1)$-diversity set and completion for $\mathcal {I}'$ by simply removing $\vec {v}$ from $M^{*}$ and *S*.

For the backward direction, consider a completion ${M'}^{*}$ of $M'=M\setminus \vec {v}$ and a $(k-1)$-diversity set $S=\{\vec {s_1},\dots ,\vec {s_{k-1}}\}$ in $M'^{*}$. Let us choose an arbitrary set *C* of $(k-1)\cdot (r+1)$ coordinates in $\vec {v}$ that all contain ${\small \square }$, and let us then partition *C* into *k*-many subsets $\alpha _1,\dots ,\alpha _k$ each containing precisely $r+1$ coordinates. Now consider the vector $\vec {v}^{*}$ obtained from $\vec {v}$ as follows:

- for each $i\in [k-1]$ and every coordinate $j\in \alpha _i$, set $\vec {v}^{*}[j]$ to the opposite value of $\vec {s_i}[j]$ (*i.e.*, $\vec {v}^*[j]=1$ if and only if $\vec {s_i}[j]=0)$;
- for every other coordinate *j* of $\vec {v}^{*}$, we set $\vec {v}^{*}[j]=\vec {v}[j]$ if $\vec {v}[j]\ne {\small \square }$ and $\vec {v}^{*}[j]=0$ otherwise.

Clearly, $M^{*}=M'^{*}\cup \{\vec {v}^{*}\}$ is a completion of *M*. Moreover, since $\vec {v}^*$ differs from each vector in *S* in at least $r+1$ coordinates, $S\cup \{\vec {v}^{*}\}$ is a *k*-diversity set in $M^{*}$. $\square$

Next, we show that instances which are sufficiently large and where each vector only “interferes with” a bounded number of other vectors are easy to solve. Technically, let

$$\begin{aligned} \zeta (k,r)=3^{(k-1)\cdot (r+1)}\cdot \sum _{\alpha \in [(k-1)\cdot (r+1)+r]}\big (\alpha ! \cdot ((k-1)\cdot 2\cdot (3(k-1)\cdot (r+1)+2r))^\alpha \big ) \end{aligned}$$

be the exact meaning of “sufficiently large” here; for brevity, note that $\zeta (k,r)\in (kr)^{\mathcal {O}(kr)}$.

#### Lemma 3

Let $\mathcal {I}=(M,k,r)$ be an instance of Pow-Hyp-IS-Completion. If $|M|\ge k\cdot \zeta (k,r)$ and $|N_{r}({\vec {v}})|< \zeta (k,r)$ for every $\vec {v}\in M$, then a *k*-diversity set in $\mathcal {I}$ can be found in polynomial time.

#### Proof

One can find a solution to $\mathcal {I}$ by iterating the following greedy procedure *k* times: choose an arbitrary vector $\vec {v}$, add it into a solution, and delete all other vectors with Hamming distance at most *r* from $\vec {v}$. By the bound on $|N_{r}({\vec {v}})|$, each choice of $\vec {v}$ will only lead to the deletion of at most $\zeta (k,r)$ vectors from *M*. Moreover, since $\delta$ measures the Hamming distance only between known entries, *any* completion of the missing entries can only increase (and never decrease) the Hamming distance between vectors. Hence, the size of *M* together with the bounded size of the Hamming neighbourhood of $\vec {v}$ guarantee that this procedure will find a solution of cardinality *k* in $\mathcal {I}$ which will remain valid for every completion of *M*. $\square$

We can now move on to the main part of the proof: a procedure which either outputs a solution outright or finds an irrelevant vector.

#### Lemma 4

Let $\mathcal {I}=(M,k,r)$ be an instance of Pow-Hyp-IS-Completion such that $|N_{r}({\vec {v}})|\ge \zeta (k,r)$ for some vector $\vec {v}\in M$ and such that each vector in *M* contains at most $(k-1)\cdot (r+1)$ ${\small \square }$’s. There is a polynomial-time procedure that finds a vector $\vec {f}\in M$ satisfying the following properties:

- (*M*, *k*, *r*) is a YES-instance if and only if $\mathcal {I}' =(M\setminus \{\vec {f}\},k,r)$ is a YES-instance, and
- A completion and diversity set for $\mathcal {I}$ can be computed from a solution and diversity set for $\mathcal {I}'$ in linear time.

#### Proof

We will begin by constructing a set system over the neighbourhood of $\vec {v}$. Let $Z=\{\,z\in [d] \;{|}\;\vec {v}[z]={\small \square }\,\}$ be the set of coordinates where $\vec {v}$ is incomplete. Clearly, since (because $|N_{r}({\vec {v}})|\ge \zeta (k,r)$)

$$\begin{aligned} |N_{r}({\vec {v}})|\ge 3^{(k-1)\cdot (r+1)}\cdot \sum _{\alpha \in [(k-1)\cdot (r+1)+r]}\big (\alpha ! \cdot ((k-1)\cdot 2\cdot (3(k-1)\cdot (r+1)+2r))^\alpha \big ) \end{aligned}$$

and $|Z|\le (k-1)\cdot (r+1)$, we can find a subset $N\subseteq N_{r}({\vec {v}})$ of vectors whose cardinality is at least $\sum _{\alpha \in [(k-1)\cdot (r+1)+r]}\big (\alpha ! \cdot ((k-1)\cdot 2\cdot (3(k-1)\cdot (r+1)+2r))^\alpha \big )$ such that all vectors in *N* are precisely the same on the coordinates in *Z*, *i.e.*, $\forall \vec {x},\vec {y}\in N: \forall z\in Z: \vec {x}[z]=\vec {y}[z]$.

Now, let *F* be a set containing 2 elements for each coordinate $j\in [d]\setminus Z$ of vectors in *M*: the element ${\small \square }_j$ and the element $D_j$. We construct a set system $\mathcal {F}$ over *F* as follows. For each vector $\vec {x}\in N$, we add a set $\hat{x}$ to $\mathcal {F}$ that contains:

- ${\small \square }_j$ if and only if $\vec {x}[j]={\small \square }$, and
- $D_j$ if and only if $\vec {x}[j]\ne {\small \square }$ and $\vec {x}[j]\ne \vec {v}[j]$.

Observe that, since $\vec {x}$ contains at most $(k-1)\cdot (r+1)$ ${\small \square }$’s by assumption and since $\vec {x}$ differs from $\vec {v}$ in at most *r*-many completed coordinates, every set in $\mathcal {F}$ has cardinality at most $(k-1)\cdot (r+1)+r$. This means that there exists $\alpha \in [(k-1)\cdot (r+1)+r]$ such that there are at least $\alpha ! \cdot ((k-1)\cdot 2\cdot (3(k-1)\cdot (r+1)+2r))^\alpha$ vectors $\vec {x}\in N$ such that $|\hat{x}|=\alpha$. This means we can apply Lemma 1 to find a sunflower $\mathcal {F}'$ in $\mathcal {F}$ of cardinality at least $(k-1)\cdot 2\Big (3(k-1)\cdot (r+1)+2r\Big )+1$; for ease of presentation, we will identify the elements of $\mathcal {F}'$ with the vectors they represent. Let $\vec {f}$ be an arbitrarily chosen vector from $\mathcal {F}'$; we claim that $\vec {f}$ satisfies the properties claimed in the lemma, and to complete the proof it suffices to establish this claim.

The backward direction is trivial: if $\mathcal {I}'$ is a YES-instance then clearly $\mathcal {I}$ is a YES-instance as well. It is also easy to observe that a completion and diversity set for $\mathcal {I}$ can be computed from a solution and diversity set for $\mathcal {I}'$ in linear time (adding a vector does not change the validity of a solution). What we need to show is that if $\mathcal {I}$ is a YES-instance, then so is $\mathcal {I}'$ (*i.e.*, $(M\setminus \{\vec {f}\}, k, r)$); moreover, this final claim clearly holds if $\mathcal {I}$ admits a solution that does not contain $\vec {f}$.

So, assume that *M* admits a completion $M^*$ which contains a *k*-diversity set $S=\{\vec {f},\vec {s_1},\dots ,\vec {s_{k-1}}\}$. Let *C* be the core of the sunflower $\mathcal {F}'$, and note that all vectors in $\mathcal {F}'$ have precisely the same content in the coordinates in *C*.

***Finding a replacement for*** $\vec {f}$

We would now like to argue that, for some completion which we will define later, $\mathcal {F}'$ contains a vector that can be used to replace $\vec {f}$ in the solution.

Let $\vec {s_i}\in S$ be an arbitrary vector. First, let us consider the case that, in *M*, $\vec {s_i}$ differs from $\vec {v}$ in more than $3(k-1)\cdot (r+1)+2r$ coordinates (*i.e.*, $\vec {v}[j]\ne \vec {s_i}[j]$ in *M* for at least $3(k-1)\cdot (r+1)+2r$ choices of *j*). Then *every* vector in $\mathcal {F}'$ will have Hamming distance greater than *r* from $\vec {s_i}$ *regardless of the completion*.

Indeed, for every vector $f'\in \mathcal {F}'$ there are at most $3(k-1)\cdot (r+1)$ coordinates *j* such that at least one of $\vec {v}[j]$, $\vec {s_i}[j]$, $\vec {f'}[j]$ is equal to ${\small \square }$, meaning that there are at least 2*r* *other* coordinates where $\vec {v}$ differs from $\vec {s_i}$ and which are guaranteed to be complete—and since $\delta (\vec {f'},\vec {v})\le r$, it must hold that $\delta (\vec {f'},\vec {s_i})>r$ (by the triangle inequality). Hence indeed every vector in $\mathcal {F}'$ must have distance at least $r+1$ from $\vec {s_i}$, and in this case we will create a set $S_i=\emptyset$ (the meaning of this will become clear later).

Now, consider the converse case, *i.e.*, that $\vec {s_i}$ differs from $\vec {v}$ in at most $3(k-1)\cdot (r+1)+2r$ coordinates. We may now extend the set system over *F* by adding a set representation of $\vec {s_i}$, specifically by adding a set $Q_i$ such that $\{{\small \square }_j,D_j\}\subseteq Q_i$ if $\vec {s_i}[j]\ne \vec {v}[j]$ (note that since $\vec {v}[j]\ne {\small \square }$, this also includes the case $\vec {s_i}[j]={\small \square }$) and otherwise $\{{\small \square }_j,D_j\}\cap Q_i=\emptyset$. Observe that $|Q_i|\le 2\cdot (3(k-1)\cdot (r+1)+2r)$, and in particular $Q_i\setminus C$ intersects with at most $2\cdot (3(k-1)\cdot (r+1)+2r)$ elements of $\mathcal {F}'$. Let $S_i$ be the set of all such elements, *i.e.*, sets in $\mathcal {F}'$ which have a non-empty intersection with $Q_i$ outside of the core (formally, these are sets of $\mathcal {F}'$ that do intersect $Q_i\setminus C$).

Observe that by the construction of $\mathcal {F}'$, there must exist at least one set in the sunflower that does not lie in any $S_i$. To conclude the proof, we will show that there is a completion $M'^*$ of $M'$ such that any arbitrarily chosen vector $\vec {f'}$ in the non-empty set $\mathcal {F}'\setminus (\{\vec {f}\}\cup \bigcup _{i\in [k-1]}S_i)$ can replace $\vec {f}$ in the *k*-diversity set *S*.

*Arguing Replaceability*

Consider a new completion $M'^{*}$ of $M\setminus \vec {f}$ obtained as follows:

- For each vector $\vec {w}\in \mathcal {F}'\setminus S$, we complete
  1. the ${\small \square }$’s in $C\cup Z$ precisely in the same way as $\vec {f}$, and
  2. for every other ${\small \square }$ at coordinate *j*, we set $\vec {w}[j]=-(\vec {v}[j]-1)$ (*i.e.*, to the opposite of $\vec {v}$ – recall that $\vec {v}[j]\ne {\small \square }$ since $j\not \in Z$); and
- all other ${\small \square }$’s in all other vectors in $M\setminus \vec {f}$ are completed in precisely the same way as in $M^{*}$.

Since $M'^{*}$ precisely matches $M^{*}$ on all vectors in $S\setminus \vec {f}$, it follows that $S\setminus \vec {f}$ is a $(k-1)$-diversity set in $M'^{*}$. Moreover, consider for a contradiction that $\delta (\vec {f'},\vec {s_i})\le r$ for some $\vec {s_i}\in S$ *after completion*, *i.e.*, in $M'^{*}$. Then clearly $\vec {s_i}$ could not differ from $\vec {v}$ in more than $3(k-1)\cdot (r+1)+2r$ coordinates in $M'$, since—as we already argued—in this case every vector in $\mathcal {F}'$ will have Hamming distance greater than *r* from $\vec {s_i}$ regardless of the completion.

Hence, we must be in the case where $\vec {s_i}$ differed from $\vec {v}$ in at most $3(k-1)\cdot (r+1)+2r$ coordinates in $M'$. Now consider how $\delta (\vec {f'},\vec {s_i})$ differs from $\delta (\vec {f},\vec {s_i})$. First of all, there is no difference between these two distances on the coordinates in $Z\cup C$ due to our construction of $M'^*$ and choice of *N*. For the remaining coordinates, we will consider separately the set *X* of coordinates in the petals of $\vec {f}$ and $\vec {f'}$ (*i.e.*, the set $\{\,j\in [d]\setminus (Z\cup C) \;{|}\;\vec {f}[j]\ne \vec {v}[j] \vee \vec {f'}[j]\ne \vec {v}[j]\,\}$), and the set $Y=[d]\setminus (C\cup Z\cup X)$ of all remaining coordinates. It follows that $\vec {v}[j]=\vec {f}[j]=\vec {f'}[j]$ for all coordinates $j\in Y$, and hence there is no difference between the two distances on these coordinates either.

So, all that is left is to consider the difference between $\delta (\vec {f'},\vec {s_i})$ and $\delta (\vec {f},\vec {s_i})$ on the coordinates in *X*; it will be useful to recall, that(unlike for the sets $Q_i$, the construction of $\mathcal {F}'$ guarantees that each coordinate occurs at most once in $\vec {f}$ and also at most once in $\vec {f'}$, and that $\alpha$ is the size of each set in the sunflower $\mathcal {F}'$. Among the coordinates in *X*, $\vec {f}$ can only differ from $\vec {s_i}$ in *at most* $\alpha -|C|$ many coordinates—notably in the coordinates of its own petal—because the coordinates in the petal of $\vec {f'}$ do not intersect with $Q_i$. On the other hand, our construction guarantees that $\vec {f'}$ differs from $\vec {s_i}$ in *at least* $\alpha -|C|$ coordinates in *X*; more precisely, on all coordinates in the petal of $\vec {f'}$, since on these coordinates (1) $\vec {s_i}$ is equal to $\vec {v}$ and (2) $\vec {f'}$ differs from $\vec {v}$.

In summary, we conclude that $\delta (\vec {f'},\vec {s_i})\ge \delta (\vec {f},\vec {s_i})$ and hence $(S\setminus \{\vec {f}\})\cup \{\vec {f'}\}$ is a *k*-diversity set in $M'^{*}$, as claimed. $\square$

We can now establish our main result for Pow-Hyp-IS-Completion.

#### Theorem 5

Pow-Hyp-IS-Completion is fixed-parameter tractable parameterized by $k+r$. In particular, Pow-Hyp-IS-Completion can be solved in time $\mathcal {O}^{*}((kr)^{\mathcal {O}(k^2r)})$, where $\mathcal {O}^{*}$ ignores polynomial factors in the runtime.

#### Proof

The algorithm proceeds as follows. Given an instance $\mathcal {I}=(M,k,r)$ of Pow-Hyp-IS-Completion, it first checks whether *M* contains a vector with more than $(k-1)\cdot (r+1)$ ${\small \square }$’s; if yes, it applies Lemma 2 and restarts on the reduced instance. Second, it checks whether $|M|\ge k\cdot \zeta (k,r)$; if not, it uses the fact that the number of ${\small \square }$’s and the number of rows is bounded by a function of the parameter to find a completion and a *k*-diversity set in $\mathcal {I}$ (or determine that one does not exist) by brute force. Note that this step can be achieved in time $$\mathcal {O}^{*}(\left( {\begin{array}{c}k\cdot \zeta (k,r)\\ k\end{array}}\right) 2^{k(k-1)\cdot (r+1)}k^{2})=\mathcal {O}^{*}((k\cdot (kr)^{\mathcal {O}(kr)})^k2^{k(k-1)\cdot (r+1)}k^{2})=\mathcal {O}^{*}((kr)^{\mathcal {O}(k^2r)})$$.

Third, it checks whether each vector $\vec {v}$ satisfies $|N_{r}({\vec {v}})|< \zeta (k,r)$; if yes, then it solves $\mathcal {I}$ by invoking Lemma 3. Otherwise, it invokes Lemma 4 to reduce the cardinality of *M* by 1 and restarts. If the algorithm eventually terminates with a “NO”, then we know that the initial input was a NO-instance; otherwise, it will output a solution which can be transformed into a solution for the original input by the used lemmas.

Since all steps, apart from solving the instance with $|M|\ge k\cdot \zeta (k,r)$ can be achieved in polynomial-time, we obtain $\mathcal {O}^*((kr)^{\mathcal {O}(k^2r)})$ as the total runtime of the algorithm. $\square$

### Lower Bounds

#### Theorem 6

Pow-Hyp-IS is NP-complete and W[1]-hard parameterized by *k*.

#### Proof

We prove both NP-hardness and W[1]-hardness results by giving a polynomial-time FPT reduction from Independent Set (IS), which is W[1]-hard [14]. Let (*G*, *k*) be an instance of IS, where $V(G)=\{v_1, \ldots , v_n\}$, and let $m=E(G)$. Fix an arbitrary ordering $\mathcal{O}=(e_1, \ldots , e_m)$ of the edges in *E*(*G*). For each vertex $v_i \in V(G)$, define a vector $\vec {a_i} \in \{0, 1\}^m$ by setting $\vec {a_i}[j]=1$ if $v_i$ is incident to $e_j$ and $\vec {a_i}[j]=0$ otherwise. Now expand the set of coordinates of these vectors by adding to each of them $n(n-1)$ new coordinates, $n-1$ coordinates for each $v_i$, $i \in [n]$; we refer to the $n-1$ (extra) coordinates of $v_i$ as the “private” coordinates of $v_i$. For each $v_i$, $i \in [n]$, set $n-1-deg(v_i)$ many coordinates among the private coordinates of $v_i$ to 1, and all other new coordinates of $v_i$ to 0. Let $M=\{\vec {a_i} \mid i \in [n]\}$ be the set of expanded vectors, where $\vec {a_i} \in \{0, 1\}^{m+n(n-1)}$, for $i \in [n]$. The reduction from IS to Pow-Hyp-IS produces the instance $\mathcal {I}=(M, k, 2n-4)$ of Pow-Hyp-IS; clearly, this reduction is a polynomial-time FPT-reduction. Observe that, for any two distinct vertices $v_i, v_j \in V(G)$, $\delta (\vec {a_i},\vec {a_j}) =2n-2$ if $v_i$ and $v_j$ are nonadjacent and $\delta (\vec {a_i},\vec {a_j}) =2n-4$ if $v_i$ and $v_j$ are adjacent. The proof that (*G*, *k*) is a Yes-instance of IS iff $(M, k, 2n-4)$ is a Yes-instance of Pow-Hyp-IS is now straightforward. $\square$

#### Theorem 7

Pow-Hyp-IS is NP-complete even when $r=2$.

#### Proof

We reduce from the Independent Set problem (which is NP-complete). Let (*G*, *k*) be an instance of Independent Set and let $G'$ be the graph obtained from *G* after subdividing every edge exactly twice. We first observe that *G* has an independent set of size at least *k* if and only if $G'$ has an independent set of size at least $|E(G)|+k$. This is because if $I \subseteq V(G)$ is an independent set of *G*, then we can add one of the subdivision vertices for every edge of *G* because *I* does not contain both endpoints of an edge. On the other hand, if $I \subseteq V(G')$ is an independent set of $G'$, then we can assume without loss of generality that *I* does not contain both endpoints of an edge in *G* because we could easily transform *I* into an independent set of the same size by replacing one of the endpoints of such an edge with a subdivided vertex. Next we construct an instance $\mathcal {I}=(M,|E(G)|+k,2)$ of Pow-Hyp-IS in polynomial-time such that $G'$ has an independent set of size at least $|E(G)|-k$ if and only if $\mathcal {I}$ is a Yes-instance. We set $d=2|V(G)|$ and obtain *M* as follows. Let $V(G)=\{v_1,\dots ,v_n\}$. For every $v_i \in V(G)$, we add the vector $\vec {v}_i$ that is 1 at the two coordinates *i* and $i+1$ and otherwise 0. Moreover, for every $e=v_iv_j\in E(G)$, we add the vector $e^1$ that is 1 at the coordinates *i*, $i+1$, and *j* and the vector $e^{2}$ that is 1 at the coordinates *j*, $j+1$, and *i*. This completes the construction of $\mathcal {I}$. The equivalence now follows because two vectors in *M* have distance at most $r=2$ if and only if their corresponding vertices in $G'$ are adjacent; here $e^{1}$ and $e^{2}$ correspond to the two subdivision vertices on the edge *e*. $\square$

## On Graph Problems on Induced Subgraphs of the Hypercubes

In this section, we discuss the implications of the results in the previous section for fundamental problems defined on induced subgraphs of powers of the hypercube graph.

In particular, the *d*-dimensional hypercube graph is the graph $Q_d$ whose vertex set is the set of all Boolean *d*-dimensional vectors, and two vertices are adjacent if and only if their two vectors differ in precisely 1 coordinate. We can then define the class $\mathcal {Q}^{r}_d$ as the class of all graphs that are induced subgraphs of the *r*-th power of $Q_d$. We note that, in line with the commonly used definition of hypercube graphs [15, 28], we consider the vertices in $\mathcal {Q}^{r}_d$ to be vectors and hence every graph $G\in \mathcal {Q}^{r}_d$ contains an explicit characterisation of its vertices as vectors. In this setting, it is straightforward to observe that Pow-Hyp-IS is precisely the Independent Set problem on $\mathcal {Q}_d^r$. Moreover, the clustering problems In-Clustering, Diam-Clustering, and Large Diam-Cluster considered in [19, 21] are precisely the Dominating Set, Partition Into Cliques, and Clique problems, respectively, on $\mathcal {Q}_d^r$. Therefore, all the upper and lower bound results derived in this paper and in [19, 21] pertaining to these clustering problems hold true for their corresponding graph problems on $\mathcal {Q}_{d}^{r}$.

### Corollary 8

Given $r,d,k \in \mathbb {N}$ and a graph $G\in \mathcal {Q}^r_d$, determining whether *G* has a:

- dominating set of size *k* is FPT parameterized by $k+r$;
- partition into *k* cliques is FPT parameterized by $k+r$;
- independent set of size *k* is FPT parameterized by $k+r$;
- clique of size *k* is FPT parameterized by *r*.

We note that all the tractability results outlined in Corollary 8 are tight, which follows from the lower-bound results obtained in Section 3.2 and in [19, 21], in the sense that dropping any parameter from our parameterizations leads to an intractable problem.

Observing that three of the graph properties in the problems discussed above are expressible in First Order Logic (FO) and result in FO formulas whose length is a function of the parameter *k*, an interesting question that ensues from the above discussion is whether these positive results can be extended to the generic problem of First-Order Model Checking [37, 44], formalised below. We will show next that the answer to this question is negative—and, in fact, remains negative even when we restrict ourselves to induced subgraphs of hypercubes (*i.e.*, for $r=1$).

**Table:** 

Q-FO-Model-Checking	
Input	A first-order (FO) formula $\Phi$, integers *d*, *r*, and a graph $G \in \mathcal {Q}_{d}^{r}$.
Parameter	$|\Phi |$
Question	Does $G \models \Phi$?

We denote by FO-Model-Checking the general FO Model Checking problem on graphs, *i.e.*, $\mathcal {C}$-FO-Model-Checking with $\mathcal {C}$ being the class of all graphs.

### Lemma 9

Let *H* be an arbitrary graph. There is a graph $G \in \mathcal {Q}^1_{|V(H)|+|E(H)|}$ such that *G* is isomorphic to the graph $H'$ obtained from *H* after subdividing every edge of *H* exactly once and attaching a leaf to every vertex resulting from a subdivision. Moreover, *G* can be computed from *H* in polynomial time.

### Proof

Let $n=|V(H)|$ and $m=|E(H)|$. To prove the lemma, we construct a matrix representation $M \in \{0,1\}^{n+m}$ of $H'$ which has one row (vector) for every vertex in *H* and where two vertices in $H'$ are adjacent if and only if their corresponding rows in *M* have Hamming distance at most 1. Let $v_1,\ldots , v_n$ be an arbitrary ordering of the vertices of *H*, and $e_1,\ldots ,e_m$ be an arbitrary ordering of its edges. Then, *M* contains one row $r_i$ for every $i \in [n]$ that is 1 at its *i*-th entry and 0 at all other entries. Moreover, for every edge $e_\ell =\{v_i,v_j\} \in E(H)$, *M* contains the following two rows:

- the row $r_e$ (corresponding to the degree-3 vertex in $H'$ obtained from *e*) that is 1 at the *i*-th and *j*-th entries, and 0 at all other entries; and
- the row $r_e'$ (corresponding to the leaf in $H'$ obtained from *e*) that is 1 at the *i*-th, *j*-th, and ($n+\ell$)-th entries, and 0 at all other entries.

This completes the construction of *M*. Clearly, two rows in *M* have Hamming distance at most one if and only if their corresponding vertices in $H'$ are adjacent, as required. $\square$

### Theorem 10

$\mathcal {Q}$-FO-model-checking is W *[t]*-hard for every $t \in \mathbb {N}^{*}$.

### Proof

We give a parameterized reduction from FO Model Checking, which is W[t]-hard for every $t \in \mathbb {N}^*$. Let $\mathcal {I}:=(\Phi ,H)$ be an instance of FO Model Checking. We will show the theorem by constructing the equivalent instance $\mathcal {I}':=(\Phi ',G)$ such that $G \in \mathcal {Q}_d^1$ and $|\Phi |\le f(|\Phi '|)$ for some computable function *f* and value *d* that is polynomially bounded in the input size. *G* is obtained from *H* in the same manner as in Lemma 9. Moreover, $\Phi '$ is obtained from $\Phi$ as follows:

- Let $\phi _V(x)$ be the formula that holds for a variable *x* if and only if *x* corresponds to one of the original vertices in *G*, *i.e.*, $\phi _V(x):=\forall y E(x,y) \exists z \ne x \wedge E(y,z)$;
- replace every subformula of the form $\exists x \phi$ (for some variable *x* and some subformula $\phi$ of $\Phi$) with the formula $\exists x \phi _{V}(x) \wedge \phi$;
- replace every subformula of the form $\forall x \phi$ (for some variable *x* and some subformula $\phi$ of $\Phi$) with the formula $\forall x \phi _V(x) \rightarrow \phi$; and
- replace every atom *E*(*x*, *y*), where *E* is the adjacency predicate and *x* and *y* are variables, with the formula $\exists s E(x,s) \wedge E(s,y)\wedge x\ne y$.

It is straightforward now to show that $H \models \Phi$ if and only if $G \models \Phi '$, and that $|\Phi '|\le 20|\Phi |$. Moreover, because of Lemma 9, $G' \in \mathcal {Q}_d^{1}$, as required. $\square$

## Conclusion

In this paper, we studied the parameterized complexity of the classical Independent Set problem on induced subgraphs of powers of hypercubes, but with the additional complication that the “positions” of the vertices in the hypercube representation may be partially unknown. We considered the two most natural parameters for the problem: the size *k* of the independent set and the power *r* of the hypercube, and provided a complete characterisation of the problem’s complexity w.r.t. *k* and *r*. We also performed a meta-investigation of the parameterized complexity of graph problems on this graph class that are expressible in FO logic and showed the existence of such problems that are parameterized intractable.

A natural future direction of our work is to study the parameterized complexity of other graph problems on this class, in particular those that have applications in clustering. One famous open problem that comes to mind is the *p*-center problem [16, 33]. The problem can be formulated similarly to the above setting, with the exception of allowing the selection of vertices to be from the whole hypercube, as opposed to restricting them to the input subgraph. In particular, the well-known *p*-centers problem reduces to the *k*-dominating set problem in the *r*-th power of the hypercube graph, but where the *k* vertices in the dominating set are not restricted to the input subgraph, but can be chosen from $\mathcal {Q}_d$. This problem was shown to be FPT parameterized by $k+r$ [21]. An intriguing NP-hard restriction of the problem is the problem slice corresponding to $p=1$, or what is known as the 1-center problem, or equivalently, the Closest String problem [33, 43]. The parameterized complexity of the problem paramertized by each of *k* and *r* alone remain important open questions.

## Data Availability

No datasets were generated or analysed during the current study.
